# Cell Fusion of Mesenchymal Stem/Stromal Cells and Breast Cancer Cells Leads to the Formation of Hybrid Cells Exhibiting Diverse and Individual (Stem Cell) Characteristics

**DOI:** 10.3390/ijms21249636

**Published:** 2020-12-17

**Authors:** Jessica Dörnen, Ola Myklebost, Thomas Dittmar

**Affiliations:** 1 Center for Biomedical Education and Research (ZBAF), Institute of Immunology, Witten/Herdecke University, Stockumer Str. 10, 58448 Witten, Germany; jessica.doernen@uni-wh.de; 2 Department of Clinical Science, University of Bergen, Postboks 7804, 5020 Bergen, Norway; Ola.Myklebost@uib.no

**Keywords:** cell fusion, cancer stem cells, EMT, hybrid clones, breast cancer

## Abstract

Cancer is one of the most common diseases worldwide, and treatment bears many challenges such as drug and radioresistance and formation of metastases. These difficulties are due to tumor heterogeneity, which has many origins. One may be cell fusion, a process that is relevant in both physiological (e.g., wound healing) and pathophysiological (cancer and viral infection) processes. In this study, we examined if cell fusion between mesenchymal stem/stromal cells (MSCs) and breast cancer (BC) cells occurs and if newly generated hybrid cells may exhibit cancer stem/initiating cell (CS/IC) characteristics. Therefore, several methods such as mammosphere assay, AldeRed assay, flow cytometry (CD24, CD44, CD104) and Western blot analysis (of epithelial to mesenchymal transition markers such as SNAIL, SLUG and Twist) were applied. In short, four different hybrid clones, verified by short tandem repeat (STR) analysis, were analyzed; each expressed an individual phenotype that seemed not to be explicitly related to either a more stem cell or cancer cell phenotype. These results show that cancer cells and MSCs are able to fuse spontaneously in vitro, thereby giving rise to hybrid cells with new properties, which likely indicate that cell fusion may be a trigger for tumor heterogeneity.

## 1. Introduction

Breast cancer (BC) is the most frequently diagnosed cancer in women, and the second most frequent overall [1]. This disease can be caused by a variety of factors concerning lifestyle, the art of living, habitation, family background and genetic variations, to name a few [1]. The complexity of BC is one reason why treatment is particularly difficult and needs multidisciplinary investigations [2]. The very fact that BC can be separated into different subtypes relating to the histology, the genetic hormone receptor status and the invasiveness underlines this aspect. Tumor heterogeneity is a huge problem, as are metastasis and recurrences. Unfortunately, there still is little known about how and why these phenomena occur.

In this context, so-called cancer stem/initiating cells (CS/ICs) came to the forefront of research. CS/ICs are thought to be the reason for cancer recurrences, but also for tumor invasion and resistance against chemotherapy and radiation treatment [3]. They are mainly distinguished by their ability to self-renew as well as to differentiate. Many hypotheses have been made about the origin of CS/ICs. It was demonstrated that different factors might be responsible for the formation of CS/ICs, including differentiation of adult stem cells into CS/ICs [4] and differentiation of non-CS/ICs into CS/ICs by mechanisms such as horizontal gene transfer, metabolic shifts and cell fusion [3]. Because of these different possible origins, CS/ICs show phenotypic plasticity leading to a heterogeneous cell population, making it difficult to define CS/IC characteristics [5]. There are also processes such as epithelial to mesenchymal transition (EMT) promoting this state [6]. In this study, we wanted to focus on cell fusion and to determine if cell fusion hybrid cells gain CSC characteristics.

Cell fusion is a rare but very important process found in physiological processes, including tissue regeneration [7], fertilization [8], and muscle development or osteogenesis [9], as well as in pathophysiological contexts such as viral infection [10]. It was demonstrated by different working groups that fused cells could be found within a solid tumor [11,12,13] and in the circulating blood [14,15,16]. These newly gained hybrid cells exhibited different characteristics than the parental cells. Xu et al. showed that hybrid cells derived from the fusion of mesenchymal stem/stromal cells (MSCs) and three different lung cancer cell lines (A549, H460 and SK-MES-1) exhibited an increased tumorigenic potential when the cells were injected into NOD/SCID mice. Fused cells gave rise to larger tumors and additionally produced tumors more efficiently than the respective parental lung cancer cells [17]. In a different study, Mi et al. demonstrated that the metastatic potential of tumor × tumor hybrid cells was increased compared to unfused, parental tumor cells [18]. Likewise, altered resistance to radiation and chemotherapy was also seen in hybrid cells [19,20]. The fusion of cancer cells with cell lines exhibiting a mesenchymal phenotype, such as leukocytes or mesenchymal stem/stromal cells, may lead to a gain of function within cancer cells, afterward exhibiting the ability to migrate.

In previous studies, we demonstrated that hybrid cells, generated by spontaneous cell fusion of human breast epithelial M13SV1-EGFP-Neo cells and different breast cancer cell lines (HS578T Hyg; MDA-MB-231 Hyg; MDA-MB-435 Hyg), exhibited individual stem cell and/or tumorigenic characteristics. M13MDA435 hybrids, for example, showed increased migratory activity and different resistance capabilities compared to the parental cell lines [7]. A variety of stem cell properties, such as the formation of mammospheres or the number of ALDH1-positive cells, had been demonstrated for M13HS hybrid cells [21]. In addition to these results, we concluded that hybrid properties were extremely dependent on parental cell characteristics [22]. To amplify this hypothesis, we generated hybrid cells by spontaneous fusion of immortalized mesenchymal stem cells (iMSC#3) and either MDA-MB-231 Hyg or HS578T Hyg breast cancer cells. iMSC#3 cells are telomerase-immortalized, nontumorigenic and can be cultured for a long time [23]. It was previously reported that MSCs could be found in primary breast cancer tissues and that these cells impact the proliferation and mammosphere formation of MCF7 cells [24]. Here, we investigated different stem cell (SC)/CS/IC characteristics such as the number of ALDH1-positive cells, the formation of colonies and mammospheres, and different markers using flow cytometry and Western blot analysis. In short, we could confirm that hybrid cells exhibit an individual phenotype and that this phenotype is dependent on parental cell line characteristics.

## 2. Results

### 2.1. Generation of Cell Fusion Hybrid Clones

Within tumor tissue, there are many different cell types, such as fibroblasts, immunocompetent cells and MSCs. To investigate if spontaneous cell fusion of MSCs and breast cancer cells could take place, in vitro studies were done. Therefore, two different human breast cancer cell lines (MDA-MB-231 Hyg and HS578T Hyg) were separately cocultured with human immortalized mesenchymal stem cells (iMSC#3). Possible hybrid clones were identified by double antibiotic selection and verified by STR-analysis (Figure S1). Four MDA-MB-231 Hyg × iMSC#3 hybrid clones (named MM#X) were analyzed, but only two could be verified as proper hybrid clones. By the fusion of HS578T Hyg × iMSC#3 cells, eleven hybrid clones were generated (named MH#X), but only 10 could be verified. For further studies, two hybrid clones from each fusion combination were selected, MM#2, MM#3, MH#7 and MH#9, because they showed the highest amount of overlapping parental alleles.

### 2.2. General Characteristics of the Hybrid Clones

**Figure 1:**
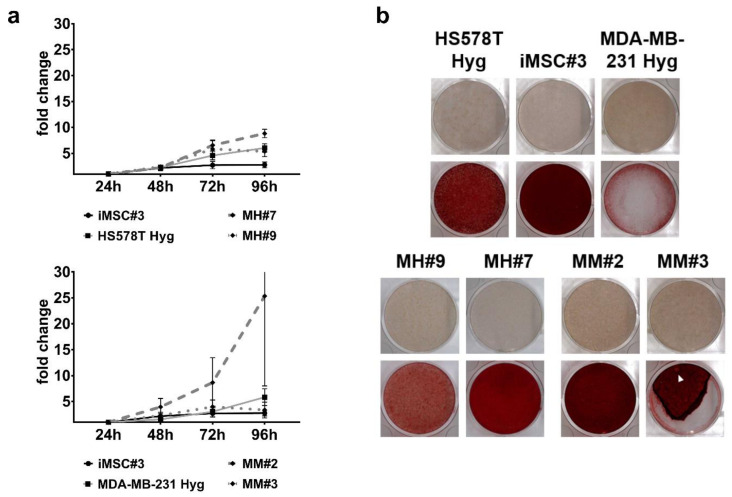
General characterization of MM and MH hybrid cells (**a**) The XTT-proliferation assay demonstrated that hybrid cells MM#2 and MH#7 exhibited a higher proliferation rate than both parental cell lines, while MM#3 and MH#9 showed an increased proliferation rate compared to iMSC#3 cells. The fold change was set to one for the data generated after 24 h. Shown are the means of at least three independent experiments. (**b**) All 4 hybrid cells were able to undergo osteogenic differentiation, as well as iMSC#3 and HS578T Hyg parental cells. A white arrow marked the formation of a knob, which was found in MM#3 cells.

Initially, general differences between the hybrid clones and their parental cell lines were examined. In an XTT-proliferation assay, the metabolic activity, which can be correlated to the proliferation of the cells, was tested. Hybrid clones MH#7 and MM#2 showed an increased proliferation rate in comparison to both parental cell lines, while MM#3 cells seemed to grow fast in the first 72 h, but then the proliferation rate decreased as fast as it first increased (Figure 1a). The large size of the cells may have had an impact on the proliferation rate; for example, the tight contact of the cells may have led to a growth arrest, or the cells had already reached the stationary phase within the growth curve. MH#9 cells proliferated faster than the iMSC#3, but slower than the HS578T Hyg cells.

Skarn et al. showed that iMSC#3 cells were able to differentiate into osteoblasts [23]. Hence, the ability to differentiate was tested for MDA-MB-231 Hyg, HS578T Hyg and all four hybrid cells. As shown in Figure 1, in addition to iMSC#3 cells, all four hybrid cells were also able to differentiate. Notably, MM#2 seemed to undergo osteogenic differentiation to a greater extent than MH#7 and MH#9. MM, #3 formed some kind of 3D structure, detached from the plate surface, and formed knobs (Figure 1b, white arrow). From the parental BC cell lines, HS578T Hyg cells also showed an enhanced ability to differentiate in comparison to MDA-MB-231 Hyg cells. In summary, the results for MDA-MB-231 Hyg and MH#9 cells showed a lighter staining intensity, indicating that they did not express stem cell characteristics in a high manner, as, for instance, MM#2 cells did.

### 2.3. Hybrid Clones Show Different and Distinct Expression Patterns of Stem Cell Characteristics

**Figure 2:**
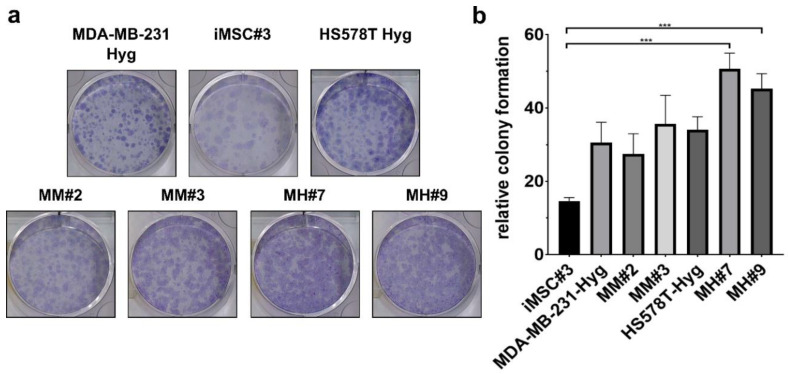
Analyses of different stem cell characteristics. (**a**) Representative images of colonies formed by the appropriate cell lines colored with crystal violet solution. (**b**) Examination of the mean relative formation of colonies. Statistical analysis was performed using a one-way ANOVA and post hoc Dunn’s multiple comparison test: *** = *p* < 0.001.

To test the clonogenic potential of the hybrid clones, a colony formation assay was performed. Hybrid clones MH#7 and MH#9 showed a significantly increased colony formation capacity in comparison to both parental cell lines. In addition, MM#2 and MM#3 cells were able to form more colonies than iMSC#3 cells (Figure 2a,b).

**Figure 3:**
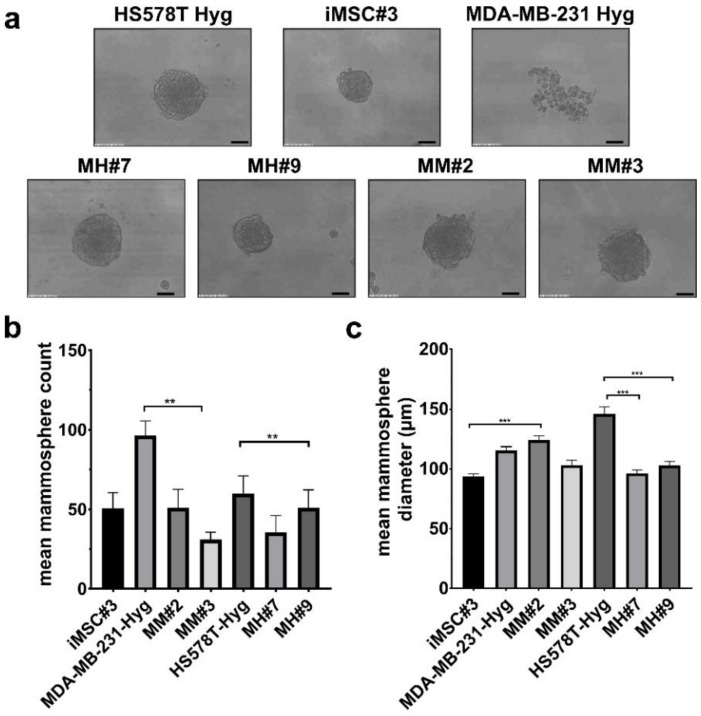
Analyses of different stem cell characteristics. (**a**) Representative images of mammospheres formed by the appropriate cell lines. Bar = 100 µm. (**b**,**c**) Shown are the mean count and diameter values of mammospheres of three independent experiments. Statistical analysis was performed using a one-way ANOVA and post hoc test: ** = *p* < 0.01, *** = *p* < 0.001.

Next, the ability to form mammospheres was tested. In comparison to iMSC#3 cells and HS578T-Hyg breast cancer cells, MDA-MB-231 Hyg cells formed loose cell aggregates rather than proper mammospheres (Figure 3a). The hybrids MM#2 and MM#3 were able to form proper mammospheres, which were larger than the iMSC#3 spheres, but did not form as many. The same applies to the hybrids MH#7 and MH#9. They showed slightly larger spheres than did iMSC#3 cells, but there were not as many. The BC cells HS578T Hyg exhibited the significantly largest and the highest amount of mammospheres of all tested cell lines (Figure 3b,c).

In summary, there seem to be correlations between the results of three in four experiments, but the constitutions differ between the single cell lines. For example, the XTT-assay, mammosphere diameter and osteogenic differentiation results demonstrate that MM#2 cells expressed these characteristics at a higher level, whereas MH#9 cells expressed them on a low level. In contrast, the CFA data show exactly the opposite result. These data indicate that hybrid cells express stem cell characteristics very individually.

**Figure 4:**
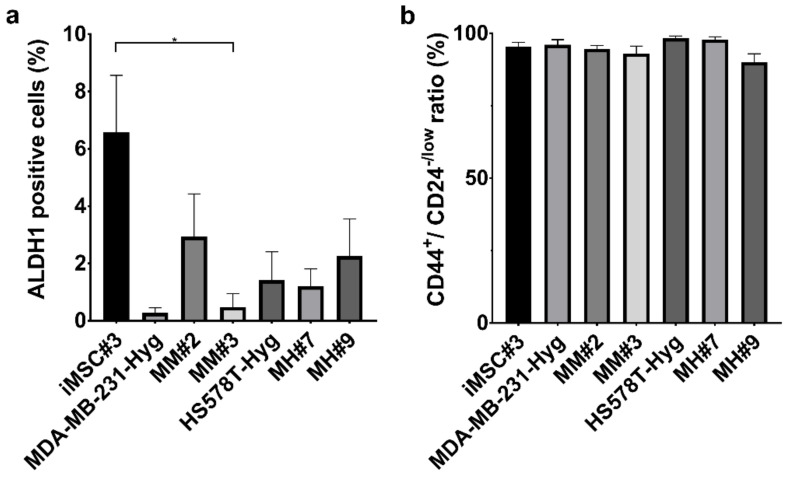
Analyses of different stem cell characteristics. (**a**) The number of aldehyde dehydrogenase 1 (ALDH1)-positive cells in the tested cell lines was examined using flow cytometry. Statistical analysis was performed using a one-way ANOVA and post hoc Tukey’s multiple comparison test: * = *p* < 0.05. (**b**) The expression of CD44 and CD24 was analyzed by flow cytometry using cells of different passages in at least three independent experiments.

By the use of the AldeRed Assay, the number of expressed ALDH1 positive cells was investigated. ALDH1 has been suggested to be a marker for adult stem cells, but also for cancer stem/initiating cells [25]. As expected, iMSC#3 cells produced the highest number of ALDH1-positive cells, approximately 6%. In contrast, virtually no ALDH1-positive MDA-MB-231 Hyg cells were detected, which is in agreement with other studies [22,26]. Interestingly, both MM#2 and MM#3 cells exhibited an increased number of ALDH1-positive cells in comparison to the MDA-MB-231 Hyg parental cell line. Analysis of MH#7 and MH#9 hybrid clones revealed that the number of ALDH1-positive cells, approximately 2%, was comparable to HS578T Hyg cells (Figure 4a).

An additional aid for identifying breast cancer stem cells is the expression of CD44^+^/CD24^−/low^ cells. The three parental cell lines and the four hybrid clones were analyzed using flow cytometry. In brief, all seven cell lines exhibited more than 90% of CD44^+^/CD24^−/low^ cells (Figure 4b).

### 2.4. EMT-Marker

**Figure 5:**
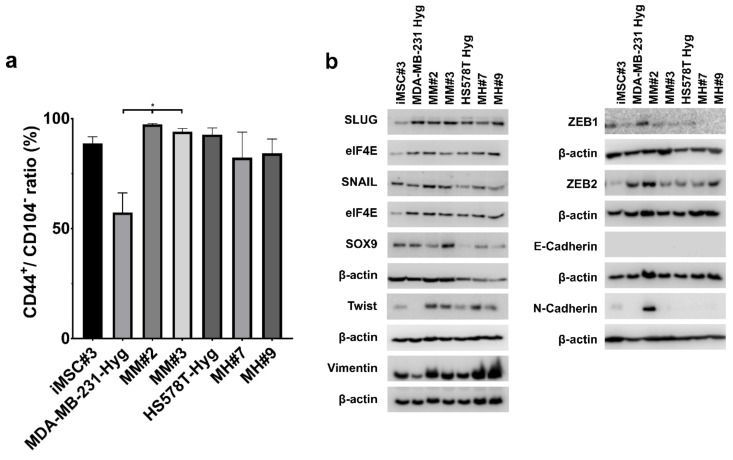
Epithelial to mesenchymal transition (EMT) markers are differentially expressed in hybrid and parental cells. (**a**) Ratio of CD44^+^ and CD104^−^ was tested by flow cytometry. Statistical analysis was performed using a one-way ANOVA test: * = *p* < 0.05. (**b**) Representative results of at least three independent Western blot analyses. The proteins SLUG, SNAIL, SOX9, Twist, vimentin, ZEB1, ZEB2, E-cadherin and N-cadherin were tested. All tested cell lines show individual expression patterns.

Epithelial to mesenchymal transition markers include SLUG, SNAIL and SOX9, as well as CD44/CD104. The expression of CD44^+^/CD104^+^ is typical for cells undergoing EMT [27]. The presence of these markers was examined using FACS analysis. For all cell lines except MDA-MB-231 Hyg, over 80% of all cells exhibited a CD44^+^/CD104^−^ expression pattern, which is characteristic of mesenchymal cells (Figure 5a). Approximately 60% of MDA-MB-231 Hyg cells were CD44^+^/CD104^−^ and 40% were CD44^+^/CD104^+^. For hybrid clones MH#7 and MH#9, approximately 15–20% of all cells were CD44^+^/CD104^+^.

In addition to CD44/CD104, additional markers were examined in Western blot studies. The parental BC cell lines MDA-MB-231 Hyg and HS578T Hyg showed a similar expression profile. Both expressed SLUG, SNAIL, vimentin and ZEB2, but lacked E-cadherin, N-cadherin and ZEB1. While MDA-MB-231 Hyg cells lacked Twist, HS578T Hyg cells lacked SOX9 expression. iMSC#3 cells expressed all of the investigated markers except E-cadherin, but mostly in small amounts. The hybrid clone MM#2 showed a similar expression profile as iMSC#3 cells. MM#3 expressed a larger amount of SNAIL, SLUG, SOX9, vimentin and Twist in comparison to both parental cell lines. Therefore, no E-cadherin, N-cadherin and ZEB1 were detectable. MH#7 and MH#9 also expressed more SNAIL, SOX9 and vimentin compared to both parental cell lines. Both MH-hybrid clones were negative for E-cadherin, N-cadherin and ZEB1 (Figure 5b).

### 2.5. Analysis of Signaling Kinetics

**Figure 6:**
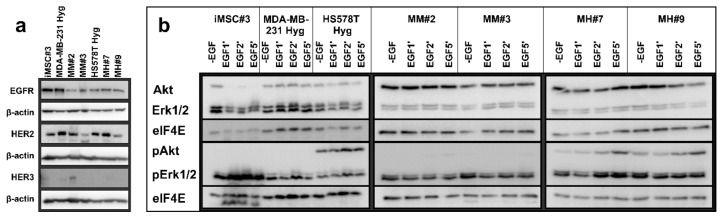
Representative images of Western blot studies. (**a**) Shown are the expression patterns of epidermal growth factor receptor (EGFR), human epidermal growth factor receptor 2 (HER2) and HER3 for all seven cell lines. (**b**) Cells were treated with EGF for 1, 2 or 5 min. Afterward, the expression of Akt, pAkt, Erk1/2 and pErk1/2 was determined by Western blot analysis.

Likewise, the expression of human epidermal growth factor receptor 2 (HER2), HER3 and epidermal growth factor receptor (EGFR) was also analyzed. While the expression of EGFR and HER2 was detectable for all cell lines, only MM#2 expressed HER3 (Figure 6a).

To analyze which signaling pathway may be active in the hybrid cells, cells were stimulated with epidermal growth factor (EGF) for 1, 2 or 5 min. Afterward, Western blot analysis was used to investigate if phosphorylated Akt and/or Erk1/2 is expressed (Figure 6b). Nonphosphorylated Akt was detectable in all cell lines except in iMSC#3 cells upon stimulation with EGF. Non-phosphorylated Erk1/2 was expressed by all cell lines, but in iMSC#3 and MH#9 cells, the amount seemed to be lower in stimulated cells compared to unstimulated cells. In iMSC#3, MDA-MB-231 Hyg, MM#2 and MM#3 cells, no phosphorylated Akt (pAkt) was detectable, while in HS578T Hyg, MH#7 and MH#9 cells, the amount of pAkt increased in accordance with EGF exposure time. Likewise, increasing amounts of phosphorylated Erk1/2 (pErk1/2) were seen in iMSC#3, HS578T Hyg and MH#7 cells, while the amount of pErk1/2 in the other cells was constant or even decreasing in accordance with the time of EGF treatment time. These results match the previous results regarding the mixed capacities hybrid cells gained from their parental cells.

### 2.6. Cytotoxicity Tests

**Figure 7:**
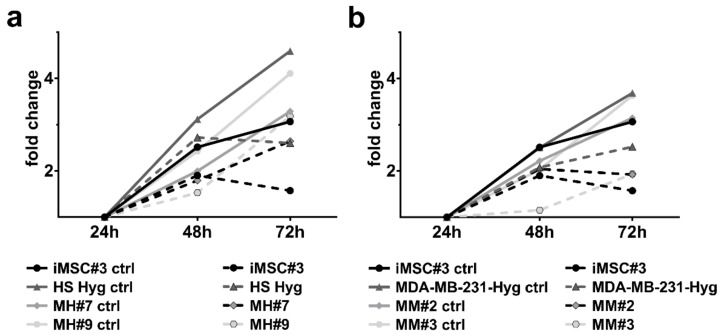
Summary of cytotoxicity test results for (**a**) iMSC#3, HS578T Hyg, MH#7, and MH#9 cells and (**b**) iMSC#3, MDA-MB-231 Hyg, MM#2 and MM#3 cells, which were treated with 0.1 µM doxorubicin. MH#7, MH#9 and MM#3 were able to grow even under cytotoxic conditions. The fold change was set to one for the data generated after 24 h. Shown are the means of at least three independent experiments. Complete data are to be found in the Supplementary Materials (Figures S2 and S3).

To test whether the hybrid clones react differently to cytostatic drugs, XTT analysis was performed using either paclitaxel or doxorubicin at concentrations of 0.1 µM/1 µM/10 µM. In brief, all cell lines died quickly after treatment with paclitaxel. Similar findings were observed after treatment with doxorubicin for iMSC#3, HS578T Hyg, and MM#2 cells. In contrast, MDA-MB-231 Hyg, MM#3, MH#7 and MH#9 cells exhibited resistance against doxorubicin at a concentration of 0.1 µM (Figure 7a,b).

## 3. Discussion

In the present study, we characterized hybrid cells obtained by the fusion of human BC cells and human MSCs. We wanted to examine whether CSC-like cells can be formed by cell fusion and if CSCs might develop certain properties during this process. We used MSCs because these cells are located in the tumor microenvironment where they are in direct contact with cancer cells and might contribute stem-like properties, i.e., inducing EMT. The tumor microenvironment affects tumor progression and cell fusion in a crucial way, enabling different cell types to come in contact and containing molecules such as cytokines, which might function as inducers for different processes such as cell fusion [28]. Even if the cancer stem cell theory and the role of cell fusion in this context had been doubted, many previous studies have demonstrated that cancer cells are able to fuse to other cells of the tumor microenvironment, such as fibroblasts [29] or macrophages [15,16,19,30]. In 1998, Rachkovsky et al. described the formation of macrophage x melanoma cell hybrids with an enhanced migration capacity [30]. In 2017, Linström et al. demonstrated that macrophage:MCF7-hybrids have a higher radioresistant capacity [19], and Clawson et al. found macrophage hybrids in the blood of melanoma and pancreatic ductal adenocarcinoma patients [15,16]. Additionally, fibroblast × melanoma hybrids had been described as exhibiting a fibroblast-like phenotype [29]. These studies demonstrate that cancer cells and cells of the tumor microenvironment are able to fuse in vivo and in vitro and give rise to hybrid cells expressing stem cell properties.

We also wanted to look for CSC properties in our hybrid cells. Therefore, we used different markers that had been described before. Dontu et al. were the first to describe the formation of mammospheres by human mammary stem/progenitor cells [31]. They elucidated the formation of nonadherent 3D structures as a characteristic of stem cells. Yousefnia et al. used three different BC cell lines to generate mammospheres and described the stemness phenotype with the use of an in vivo chick embryo model. All mammospheres showed increased proliferation, migration and drug resistance potential and higher tumorigenicity compared to the corresponding cell lines [32]. In this study, mammospheres were formed by all four hybrid clones, HS578T Hyg and iMSC#3 cells, but not by MDA-MB-231 Hyg cells (Figure 3). MDA-MB-231 and other common breast cancer cell lines, which do not express E-cadherin, form cell clumps in sphere-forming assays [33]. If the mammospheres also show different tumorigenic potential, this finding must be evaluated in further studies. Additionally, it must be clarified why MSCs formed fewer and smaller mammospheres, as HS578T Hyg cells did. This finding likely indicates that putative cancer stem-like cells proliferate faster than MSCs.

In addition to the formation of mammospheres, ALDH1-positive cells, and a CD44^+^/CD24^−/low^ phenotype are distinctive for stem-like cell lines [25,34,35,36,37]. In our study, we were able to demonstrate that approximately 90% of the cells per cell line expressed a CD44^+^/CD24^−/low^ phenotype (Figure 4). Differences were found in the expression of ALDH1. The hybrids MM#2, MM#3 and MH#9 showed an increased number of these cells compared to the BC parental cells (Figure 4). However, a relationship between the number of ALDH1-positive cells, CD44^+^/CD24^−/low^ cells and the formation of mammospheres was not detectable for the tested cell lines. Each hybrid cell line possessed individual capabilities, which seem to not have any correlation. This result confirms what was shown in our previous study, which also found no correlation between the number of ALDH1-positive cells and the formation of mammospheres using M13SV1-EGFP-Neo and different BC cell line hybrids [22]. It also must be discussed whether stem cells truly exhibit a stable phenotype or if they rather are in a state of the permanent shift [38]. This would make it more difficult to identify them, and standard methods may be revised [39].

Besides these studies, others demonstrated that the fusion of cancer cells with other cells led to hybrid cells with an increased tumorigenic and metastatic potential and an altered drug-/radioresistance capacity [40,41,42,43]. To test whether similar propositions can be made for our hybrid cells, we analyzed their proliferation rate, their capacity to form colonies and their behavior under cytotoxic conditions. We demonstrated that MM#2 and MH#7 had an increased proliferation rate compared to both parental cell lines (Figure 1). On the other hand, the colony-forming capacity was increased for all hybrid cells compared to iMSC#3 cells (Figure 2). Under low cytotoxic conditions (0.1 µM), only MH#7 and MH#9 showed a slightly increased resistance against doxorubicin. For other concentrations and paclitaxel, no resistance potential was detectable (Figure 7). Whether our tested hybrid cells truly are more tumorigenic than the BC cell lines must be tested with in vivo studies.

Furthermore, it is unclear which impact epithelial to mesenchymal transition (EMT), and its reversal mesenchymal to epithelial transition (MET) might have on tumor progression. The transition of an epithelial to a mesenchymal phenotype is induced by different transcription factors such as SLUG, SNAIL, TWIST, ZEB1 and ZEB2 [44,45,46], leading to the loss of E-cadherin, which is necessary for cell–cell adhesion. Cells executing EMT are able to enter the bloodstream, where they might be able to exist as circulating tumor cells or undergo MET to enter into the tissue of a distant organ. Kröger et al. determined a so-called hybrid E/M state, which is characterized by the expression of CD44^hi^/CD104^+^ cells [27]. Cells in this state are highly tumorigenic. Our analyzed hybrid populations exhibited a mesenchymal phenotype in over 80% of the cells. For the MDA-MB-231 Hyg cell line, approximately 40% of the cells expressed a hybrid E/M state. More difficult is the analysis of our Western blot data. Transcription factors were expressed differently in all tested cell lines. SLUG, SNAIL, vimentin and ZEB2 are highly expressed in all cell lines, while the expression of SOX9, Twist and ZEB1 was observed to be less or only in distinct cell lines. E-cadherin was not expressed in any cell line, and N-cadherin—a mesenchymal marker—was only expressed in iMSC#3 and MM#2 cells, which is remarkable compared to the flow cytometry results (Figure 5).

In summary, in this study, we tested the mesenchymal/cancer stem cell characteristics of four hybrid cell clones in various experiments. There were no obvious correlations between distinct properties; rather, all hybrid cells exhibited individual characteristics that were not predictable. Our results indicate that cell fusion leads to the formation of a heterogenic cell population with a variety of capabilities. These results confirm a plurality of studies describing the same results for cell fusion hybrid cells [7,14,22,47,48]. However, it is unclear whether the generated hybrid cells are more tumorigenic than parental cancer cells. In a recent study, Melzer et al. demonstrated that MDA-hyb3 cells generated by the fusion of MSC and MDA-MB-231 cells showed a reduced tumor formation rate compared to MDA-MB-231 cells when injected subcutaneously in both flanks of NOD/SCID mice [49]. The number of hybrid cells that is necessary in vivo to show an effect in tumor progression remains to be elucidated, which differs in comparison to tumors in which no cell fusion events took place. These effects may be a higher metastatic or proliferation rate. There are studies demonstrating that cell fusion events take place in vivo and play a role in tumor progression.

In the early 2000s, cell fusion hybrids were discovered in patients who had previously had a bone marrow transplantation (BMT) [50,51]. Gast et al. were able to detect fusion hybrid cells in biopsies of female pancreatic ductal adenocarcinoma patients who previously received a sex-mismatched bone marrow transplant. They identified a Y-chromosome in epithelial cancer cells. Furthermore, they also identified fusion hybrids in the peripheral blood [14]. LaBerge et al. demonstrated that hybrid cells are also able to metastasize in vivo. Therefore, they analyzed primary melanoma cells and cells of the nodal metastasis of a patient who previously received a BMT and discovered alleles of both the patient and the BMT donor via STR-analysis [52].

Tumor heterogeneity remains one of the biggest problems cancer therapy must face. Many studies already demonstrated the diversity of fusion partners that cancer cells must create variegated tumor tissues, making it difficult to find the right treatment for all these cell subtypes. In the future, new starting points must be considered for fighting against this leading disease.

## 4. Materials and Methods

### 4.1. Cell Culture

- iMSC#3 cells [23] were cultured in Alpha Minimum Essential Medium (αMEM) Eagle (Pan Biotech, Aidenbach, Germany) medium supplemented with 10% fetal calf serum (FCS; Biochrom GmbH, Berlin, Germany), 100 U/mL penicillin, 0.1 mg/mL streptomycin (Sigma-Aldrich, Taufkirchen, Germany) and 2 µg/mL puromycin (InvivoGen, Toulouse, France).
- MDA-MB-231 Hyg human breast cancer cells (HTB-26; LGC Standards GmbH, Wesel, Germany) were cultured in Dulbecco’s Modified Eagle’s Medium (DMEM) (Pan Biotech, Aidenbach, Germany) medium supplemented with 10% fetal calf serum (FCS; Biochrom GmbH, Berlin, Germany), 100 U/mL penicillin, 0.1 mg/mL streptomycin (Sigma-Aldrich, Taufkirchen, Germany) and 200 µg/mL hygromycin.
- HS578T Hyg human breast cancer cells (HTB-126; LGC Standards GmbH, Wesel, Germany) were cultured in RPMI 1640 (Pan Biotech, Aidenbach, Germany) medium supplemented with 10% fetal calf serum (FCS; Biochrom GmbH, Berlin, Germany), 100 U/mL penicillin, 0.1 mg/mL streptomycin (Sigma-Aldrich, Taufkirchen, Germany) and 200 µg/mL hygromycin.
- MM-Hybrids were cultured in DMEM (Pan Biotech, Aidenbach, Germany) medium supplemented with 10% fetal calf serum (FCS; Biochrom GmbH, Berlin, Germany), 100 U/mL penicillin, 0.1 mg/mL streptomycin (Sigma-Aldrich, Taufkirchen, Germany), 1 µg/mL puromycin and 100 µg/mL hygromycin. MH-Hybrids were cultured in RPMI 1640 (Pan Biotech, Aidenbach, Germany) medium supplemented with 10% fetal calf serum (FCS; Biochrom GmbH, Berlin, Germany), 100 U/mL penicillin, 0.1 mg/mL streptomycin (Sigma-Aldrich, Taufkirchen, Germany), 1 µg/mL puromycin and 100 µg/mL hygromycin. All cells were maintained in a humidified atmosphere with 5% CO_2_ at 37 °C.

### 4.2. Spontaneous Cell Fusion

To generate cell fusion hybrids, either iMSC#3 (1 × 10^6^) and HS578T Hyg (1 × 10^6^) or iMSC#3 (1 × 10^6^) and MDA-MB-231 Hyg (1 × 10^6^) cells were co-cultivated in media specific for the appropriate breast cancer cell line at 5% CO_2_ and 37 °C. After 24 h, media were replaced by media containing antibiotics (100 µg/mL hygromycin B (AppliChem GmbH, Darmstadt, Germany); 1 µg/mL puromycin (InvivoGen, Toulouse, France)). Hybrid cells exhibiting double resistance were isolated and individually cultivated. Overall, 11 iMSC#3 × HS578T Hyg hybrids, named MH#X, and 2 iMSC#3 × MDA-MB-231 Hyg hybrids, namely, MM#X, were isolated (X = clone number).

### 4.3. STR-Analysis

Short tandem repeat analysis was performed by the working group of W. Gerding (Ruhr-University Bochum, Department of Human Genetics) using the Powerplex^®^ 16HS System DC2101 (Promega). Therefore, 100 ng genomic DNA was prepared using the NucleoSpin^®^ tissue kit (Macherey-Nagel GmbH and Co. KG, Düren, Germany) per the manufacturer’s instructions (concentration was photometrically determined) and sent to their laboratory. Examination of the degree of the relationship was performed using Excel.

### 4.4. Osteogenic Differentiation

For osteogenic differentiation, 1 × 10^5^ cells/well were seeded into a 6-well plate and incubated at 5% CO_2_ and 37 °C for 1 day. On the next day, media were exchanged. Control cells received the cell-line-specific medium, while the tested cells got osteogenic differentiation medium consisting of DMEM (Pan Biotech, Aidenbach, Germany) supplemented with 10% FCS (FCS; Biochrom GmbH, Berlin, Germany), 100 U/mL penicillin, 0.1 mg/mL streptomycin (Sigma-Aldrich, Taufkirchen, Germany), 100 nM dexamethasone 21-phosphate disodium salt (Sigma-Aldrich, Taufkirchen, Germany), 10 mM β-glycerol phosphate (Merck, Millipore, Darmstadt, Germany) and 50 µM L-ascorbic acid 2-phosphate sesquimagnesium salt hydrate (Sigma-Aldrich, Taufkirchen, Germany). The medium was replaced two times per week, and cells were cultured for 21 days at 5% CO_2_ and 37 °C. Afterward, cells were fixed in 4% paraformaldehyde (Thermo Scientific, Waltham, MA) and stained with Alizarin Red S (Sigma-Aldrich, Taufkirchen, Germany) for 30 min at RT and washed 3 times with aqua destillata (A. dest.).

### 4.5. XTT Proliferation and Cytostatic Assay

On the first day, 2500 cells/well were seeded on a 96-well plate and cultured in a cell-type-specific medium. For proliferation studies, the medium was removed after 24 h and replaced by RPMI 1640 without phenol red (Pan Biotech, Aidenbach, Germany). Then, 50 µg/well XTT and 0.25 µg/well PMS (AppliChem GmbH, Darmstadt, Germany) were added, and plates were incubated for 3 h at 37 °C. After 3 h, the reaction was measured with an ELISA reader (BioTek Instruments, Inc., Winooski, VT, USA). This procedure was repeated after 48 h, 72 h and 96 h. For cytostatic studies, the medium was also removed after 24 h, and it was replaced by a medium containing either 0.1 µmol, 1 µmol or 10 µmol doxorubicin or paclitaxel (both: Abcam, Cambridge, UK). Afterward, cells were again incubated for 24 h before media were exchanged by RPMI 1640 without phenol red (Pan Biotech, Aidenbach, Germany). Then, 50 µg/well XTT and 0.25 µg/well PMS were added, and plates were incubated 3 h at 37 °C. After 3 h, the reaction was measured with an ELISA (BioTek Instruments, Inc., Winooski, VT, USA) reader. This procedure was repeated after 48 h and 72 h.

Colony formation assay:

In a 6-well plate, 200 cells/well were seeded and cultured for 10 days, changing the cell-line-specific media after 2–3 days. After 10 days, the media were removed, and the cells were washed with PBS (Pan Biotech, Aidenbach, Germany). For fixation, 4% paraformaldehyde (Thermo Scientific, Waltham, MA) was used; for coloring of the cells, 0.5% crystal violet (Sigma-Aldrich, Taufkirchen, Germany) was used. Crystal violet was incubated for 30 min at RT and afterward washed away with H_2_O. The plate was air-dried at RT. Densitometric analysis was performed using ImageJ (imagej.-nih.gov/ij/).

### 4.6. Mammosphere Assay

For mammosphere assays, 96-well plates were coated with 1.2% poly(2-hydroxyethyl-methacrylate) (poly-HEMA) (Sigma-Aldrich, Taufkirchen, Germany) in ethanol and dried for 10 days at 37 °C. Five hundred cells/well were seeded and cultured in mammosphere formation medium at 37 °C for 7 days. Mammosphere formation medium consisted of two different media (medium I and II) in a ratio of 1:4. Medium I was composed of DMEM/F12 (Pan Biotech, Aidenbach, Germany) + B27 supplement (Gibco, Thermo Scientific, Waltham, MA) + 20 ng/mL FGF (human recombinant; Sigma-Aldrich, Taufkirchen, Germany) + 20 ng/mL EGF (human recombinant; Sigma-Aldrich, Taufkirchen, Germany) + 0.39 µg/mL hydrocortisone (Sigma-Aldrich, Taufkirchen, Germany). Medium II is composed of Methocult H4100 (Stem Cells Technologies, Köln, Germany) + DMEM (Pan Biotech, Aidenbach, Germany) (ratio 2:3). After 7 days, images were generated with a microscope and analyzed using ImageJ 1.8.0 (https://imagej.nih.gov/ij/download.html).

### 4.7. AldeRed Assay

The expression of aldehyde dehydrogenase 1 (ALDH1) was examined by using the AldeRed ALDH detection assay (Merck Millipore, Darmstadt, Germany) according to the manufacturer’s manual. First, 2 × 10^5^ cells were harvested and resuspended in AldeRed assay buffer containing the efflux inhibitor Verapamil and AldeRed 588A substrate. One half of the cell suspension was transferred to a new reaction tube and provided with diethylamino-benzaldehyde (DEAB), which is a specific ALDH1 inhibitor; this sample served as a control. Afterward, cells were incubated for 45 min at 37 °C in the dark and centrifuged (300× *g*, 5 min). The cell pellet was washed in 500 µL of AldeRed assay buffer. Samples were analyzed using fluorescence-activated cell sorting (FACSCalibur; Becton Dickenson, Heidelberg, Germany), and FACS data were evaluated using WinMDI 2.9.

### 4.8. Flow Cytometry

Flow cytometry was used to analyze CSC and EMT markers in hybrid and parental cells. In this experiment, 1.5 × 10^5^ cells were stained with different antibodies in accordance with the manufacturer’s instructions and measured with a FACScalibur flow cytometer (Becton Dickenson, Heidelberg, Germany). Isotype-matched antibodies served as controls. All antibodies are listed in Table 1.

### 4.9. Western Blot

For the generation of Western blot samples, 2 × 10^5^ cells were treated with 3× Lemmli sample buffer (ratio 1:2) and lysed for 10 min at 95 °C. EGF-stimulated samples were treated with 100 ng/mL EGF (human recombinant; Sigma-Aldrich, Taufkirchen, Germany) for 1, 2 or 5 min before 3× Lemmli sample buffer was added and cells were lysed for 10 min at 95 °C. For 8%, 10% or 12% sodium dodecyl sulfate-polyacrylamide gel electrophoresis (SDS–PAGE), the samples were separated and afterward transferred to an Immobilon polyvinyl difluoride (PVDF) nitrocellulose membrane (Merck Millipore, Darmstadt, Germany) under semidry conditions. The membranes were then blocked with 5% (*w*/*v*) nonfat milk powder in Tris-buffered saline with 1% Tween-20 (TBS-T) for at least 1 h. All antibodies were used in accordance with the manufacturer’s instructions. Visualization of protein bands was executed by using the Pierce ECL Western blot substrate (Thermo Fisher Scientific, Bonn, Germany) and the Aequoria macroscopic imaging system (Hamamatsu Photonics Germany, Herrsching am Ammersee, Germany). All images were evaluated using ImageJ (imagej.-nih.gov/ij/).

### 4.10. Statistical Analysis

Statistical analysis was performed using GraphPad Prism 7. In the case of a rectangular distribution of the data, a one-way ANOVA test with the following post hoc test was used. If not, a Kruskal-Wallis test and appropriate post hoc test were performed. Unless otherwise mentioned, data were generated in at least three independent experiments.

## Figures and Tables

**Table 1:** List of all antibodies used for flow cytometry and Western blot analyses.

Antibody	Manufacturer
CD24-PE; mouse; #ML5	BD Biosciences ^1^
CD44-APC; mouse; #G44-26	BD Biosciences ^1^
CD104-PE; rat; #439-9B	BioLegend ^2^
Akt; rabbit monoclonal; #11E7	Cell Signaling ^3^
β-actin; mouse monoclonal; #AC-74	Sigma ^4^
E-cadherin; rabbit monoclonal; #24E10	Cell Signaling ^3^
EGFR; rabbit monoclonal; #C74B9	Cell Signaling ^3^
elF4E; rabbit polyclonal	Cell Signaling ^3^
IgG2Aκ-PE; mouse; #G155-178	BD Biosciences ^1^
IgG2bκ-APC; mouse; #27-35	BD Biosciences ^1^
HER2/ErbB2; rabbit monoclonal; #29D8	Cell Signaling ^3^
HER3/ErbB3; rabbit monoclonal; #D22C5	Cell Signaling ^3^
N-cadherin; mouse; #32	BD Biosciences ^1^
p44/42 MAPK (Erk1/2); rabbit polyclonal	Cell Signaling ^3^
Phospho-Akt (Ser473) XP^®^; rabbit monoclonal; #D9E	Cell Signaling ^3^
Phospho-p44/42 MAPK (Erk1/2) (Thr202/Tyr204); rabbit polyclonal	Cell Signaling ^3^
SLUG; rabbit monoclonal; #C19G7	Cell Signaling ^3^
SNAIL; rabbit monoclonal; #C15D3	Cell Signaling ^3^
SOX9; rabbit monoclonal; #D8G8H	Cell Signaling ^3^
Twist; mouse monoclonal; #Twist2C1a	Abcam ^5^
Vimentin; rabbit; #R28	Cell Signaling ^3^
ZEB1; rabbit monoclonal; #D80D3	Cell Signaling ^3^
ZEB2; rabbit polyclonal	Abcam ^5^

^1^ BD Biosciences, Heidelberg, Germany; ^2^ BioLegend, San Diego, CA; ^3^ Cell Signaling Technology Europe B.V., Frankfurt am Main, Germany; ^4^ Sigma-Aldrich, Taufkirchen, Germany; ^5^ Abcam, Cambridge, UK.

## Supplementary Materials

Supplementary materials can be found at https://www.mdpi.com/1422-0067/21/24/9636/s1.

## Abbreviations

ALDH1	Aldehyde dehydrogenase 1
BC	Breast cancer
BMT	Bone marrow transplant
BSA	Bovine serum albumin
CSC	Cancer stem cell
EMT	Epithelial to mesenchymal transition
FCS	Fetal calf serum
HepG2	Hepatocellular carcinoma cells
MET	Mesenchymal to epithelial transition
MSC	Mesenchymal stem cell
PVDF	Polyvinyl difluoride
SC	Stem cell
SDS–PAGE	Sodium dodecyl sulfate-polyacrylamide gel electrophoresis
STR	Short tandem repeat
TBS-T	Tris-buffered saline with 1% (*v*/*v*) Tween-20
