# Supplementary material for: Cell Fusion of Mesenchymal Stem/Stromal Cells and Breast Cancer Cells Leads to the Formation of Hybrid Cells Exhibiting Diverse and Individual (Stem Cell) Characteristics
Source: Int J Mol Sci. 2020 Dec 17;21(24):9636. doi: 10.3390/ijms21249636 (PMC7765946; doi:10.3390/ijms21249636)
Supplement: Supplementary file 1 [file ijms-21-09636-s001.pdf]

Supplementary Materials:

Table S1. Genotypic analysis of MM- and MH-hybrid cells via short tandem repeat analysis.

Locus	iMSC#3	HS578T Hyg	MH#7	MH#9
D3S1358	128	128	129	129
TH01[1]	170		170	170
	174	174	174	174
		177	177	177
D21S11	215		215	216
		220	219	220
	223		223	223
		234	233	233
D18S51	306		307	307
		314	314	314
	326		326	326
Penta E	413		413	413
		418	418	418
	424	424	424	423
D5S818	127		127	127
		131	131	131
	135		135	135
D13S317	176		176	176
		188	188	188
	200		200	200
D7S820	224		224	224
	228	228	228	228
D16S539	283		283	283
	287		287	287
		291	291	291
CSF1PO[2]	338		338	338
	346	346	346	346
Penta D		394	394	394
	399		399	399
	413		413	413
		418	418	418
Am	104	104	104	104
	110		110	110
vWA[3]	138		138	139
	146		146	146
		150	150	150
D8S1179	222		222	222
		226	226	226
	230		230	230
TPOX[4]	269	269	269	269
	281		281	281
FGA[5]	337		337	337
	346		346	346
		350	350	350

Locus	iMSC#3	MDA-MB-231 Hyg	MM#2	MM#3
D3S1358	128	129	128	128
TH01[1]		166	166	166
	170		170	170
	174		174	174
		177	177	177
D21S11	215		216	216
	223	224	223	223
		238	238	238
D18S51		295	295	295
	306		307	307
		314	314	314
	326		326	326
Penta E		408	408	408
	413		413	413
	424		423	423
D5S818	127		127	127
	135	135	135	135
D13S317	176		176	176
		196	196	196
	200		200	200
D7S820		221	220	220
	224	224	224	224
	228		228	228
D16S539	283		283	283
	287		287	287
		291	291	291
CSF1PO[2]	338		338	338
		342	342	342
	346	346	346	346
Penta D	399		399	399
		408	408	408
	413		413	413
		422	422	423
Am	104	104	104	104
	110		110	110
vWA[3]	138		138	138
		142	142	142
	146		146	146
		158		158
D8S1179	222		222	222
		226	226	226
	230		230	230
TPOX[4]	269	269	269	269
		273	273	273
	281			281
FGA[5]	337		337	337
	346	346	346	346
		350	350	35

[1]TH01: human tyrosine hydrolase; [2]CSF1PO: human c-fms proto-oncogene for CSF-1 receptor; [3]vWA: human von Willebrand factor; [4]TPOX: human thyroid peroxidase; [5]FGA: human alpha fibrinogen

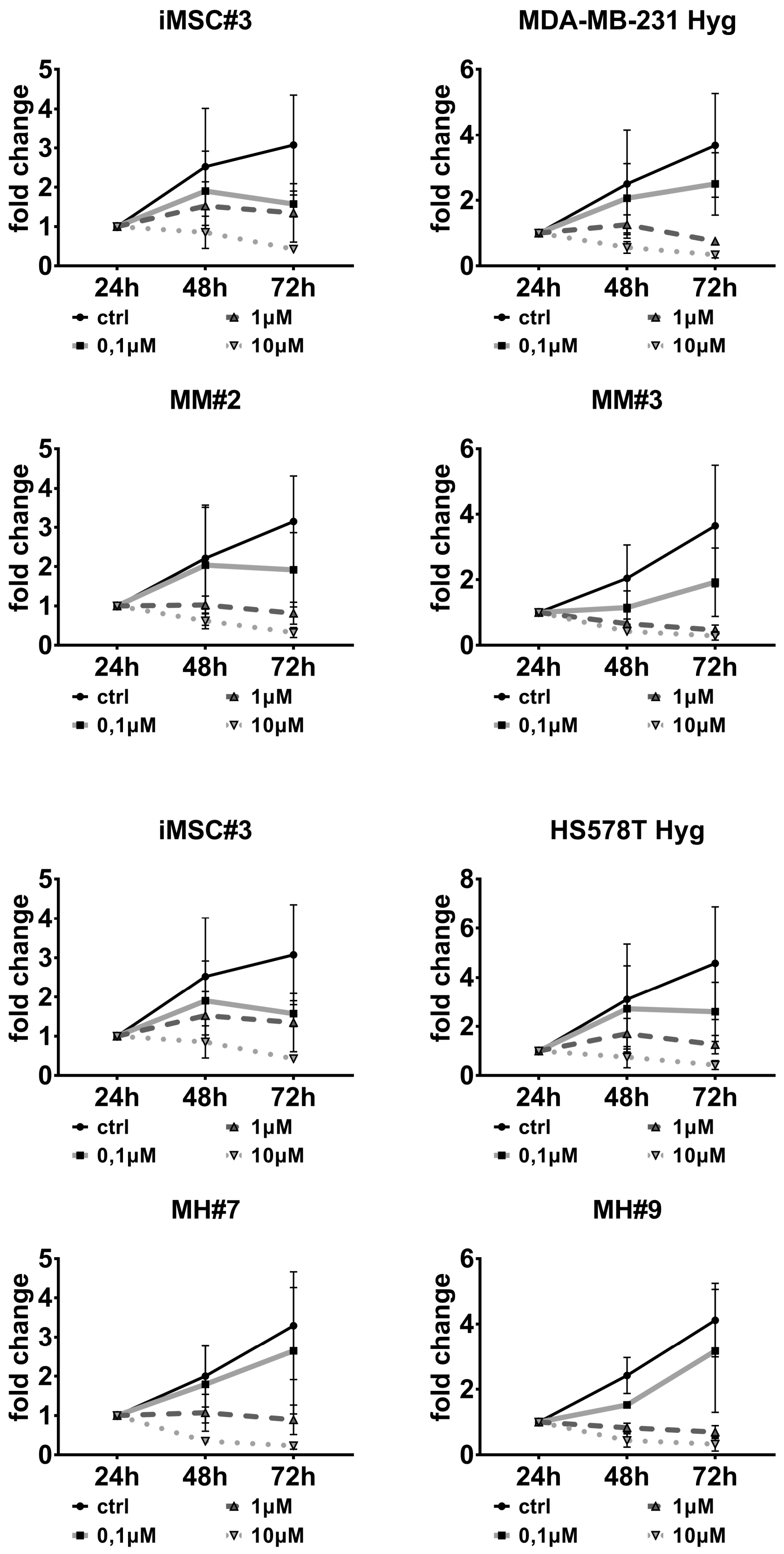

Figure S1. Cytotoxicity tests with Doxorubicin demonstrated, that hybrid cells MH#7, MH#9 and MM#3 exhibited a resistance against 0,1µM Doxorubicin. Shown are the means (with SEM) of three independent XTT-assays.

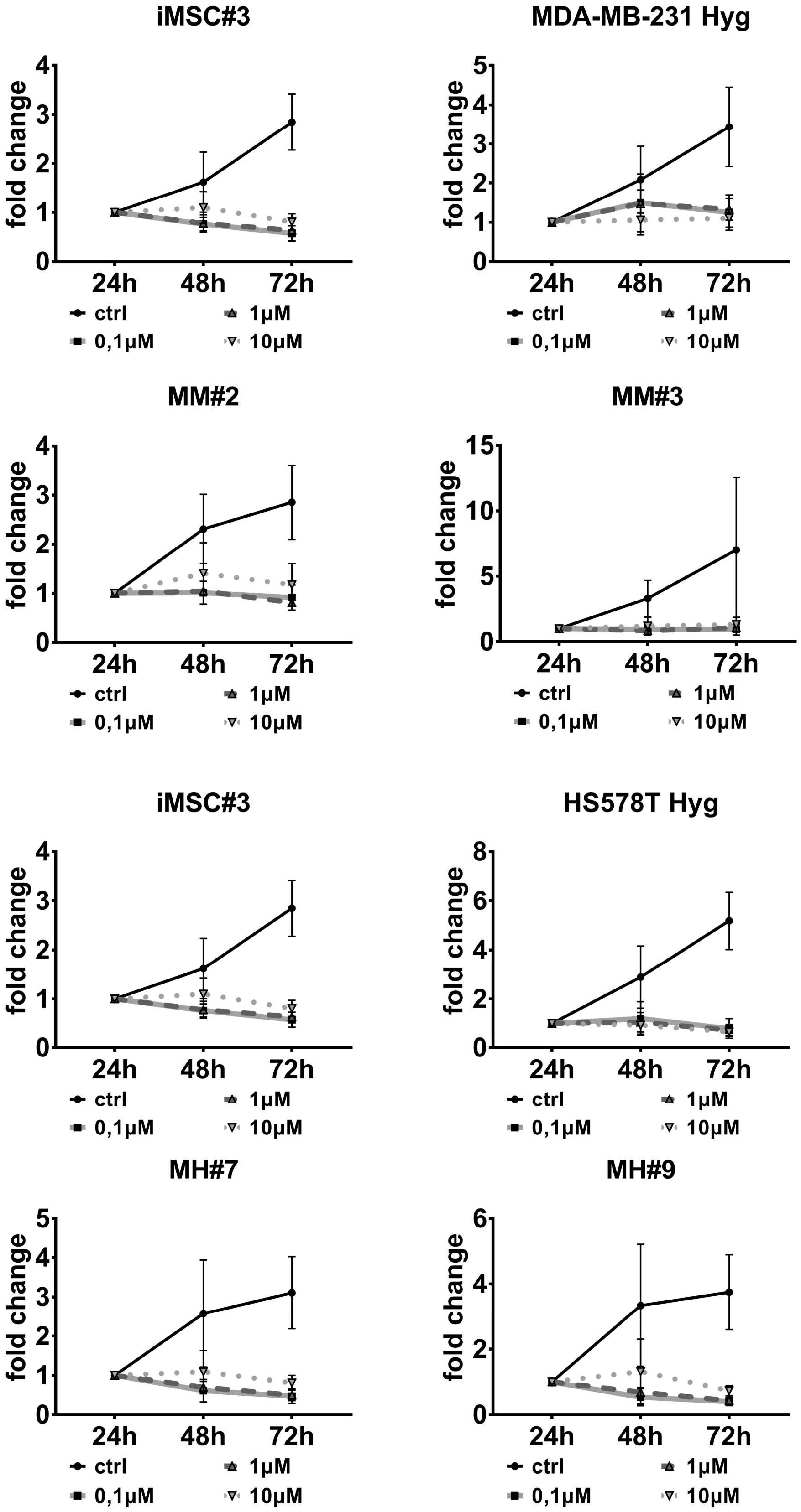

Figure S2. XTT-analysis of cells treated with different concentrations (0,1/ 1/ 10µM) of Paclitaxel. In contrast to the results for Doxorubicin, treatment with Paclitaxel led to the death of nearly all cells. Shown are the means (with SEM) of three independent experiments.
